# Validation of HPLC Method for Analysis of Gamma-Aminobutyric and Glutamic Acids in Plant Foods and Medicinal Plants

**DOI:** 10.3390/molecules28010084

**Published:** 2022-12-22

**Authors:** Daniela Pencheva, Desislava Teneva, Petko Denev

**Affiliations:** Laboratory of Biologically Active Substances, Institute of Organic Chemistry with Centre of Phytochemistry, Bulgarian Academy of Sciences, 139 Ruski Blvd., 4000 Plovdiv, Bulgaria

**Keywords:** gamma-aminobutyric acid, glutamic acid, HPLC, plant foods, medicinal plants

## Abstract

Gamma-aminobutyric acid (GABA) is the major inhibitory neurotransmitter in the central nervous system of mammals and plays an important role in the suppression of neurons’ excitability. GABA is formed from the decarboxylation of glutamic acid (Glu), and both GABA and Glu could be considered as important biologically active food components. In the current study, we validated a HPLC method for concomitant detection of GABA and Glu in plant samples after derivatization with dansyl chloride. The validated method had high precision and a high recovery rate and was successfully used for GABA and Glu quantification in 55 plant foods (fruits, vegetables, legumes, cereals, pseudocereals, and nuts) and 19 medicinal plants. Vegetables were the most important dietary source of these amino acids, with the highest quantity of GABA found in potatoes—44.86 mg/100 g fresh weight (FW) and yellow cherry tomatoes—36.82 mg/100 g FW. The highest amount of Glu (53.58 mg/100 g FW) was found in red cherry tomatoes. Analyzed fruits were relatively poor in GABA and Glu, and European gooseberry was the richest fruit with 13.18 mg/100 g FW GABA and 10.95 mg/100 g FW Glu. Cereals, pseudocereals, nuts, and legumes contain much higher amounts of Glu than GABA. The obtained results enrich the available information on the content of gamma-aminobutyric and glutamic acids in plant foods and could be used for the development of GABA-enriched functional foods.

## 1. Introduction

Gamma-aminobutyric acid (GABA) is an amino acid that functions as the primary inhibitory neurotransmitter in the central nervous system [1]. It is a four-carbon non-protein amino acid, formed by the decarboxylation of glutamic acid (Glu) through the enzyme glutamate decarboxylase. In mammals, GABA exhibits numerous health benefits related to improving sleep, reducing mental and physical stress, increasing immunity under stressful conditions, delaying signs of aging, strengthening blood vessels, and preventing obesity. It is used to treat schizophrenia, epilepsy, Parkinson’s disease, and other neurological diseases [2,3,4,5,6]. In addition, GABA exhibits hypotensive and sedative effects and is involved in the regulation of blood pressure and cholesterol levels [6]. Initially, it was believed that exogenous GABA does not cross the blood-brain barrier [7]. However, recent research suggests that this is possible, and exogenous GABA (taken with the diet or as dietary supplements) can exert GABAergic effects on the enteric nervous system, which in turn stimulates the production of endogenous GABA [8]. That is why there is enormous interest from science and industry in the development of GABA-enriched functional foods and food supplements, and it is not surprising that numerous plant foods have been investigated for their GABA content [9,10]. In addition, different biotechnological approaches have been developed in order to increase the GABA content of plant foods [11,12].

In Europe, GABA is used as an ingredient in food supplements. However, in 2009 EFSA concluded that a cause-and-effect relationship had not been established between the intake of GABA and cognitive functions. In the USA, GABA as an amino acid meets the definition of a dietary ingredient and is available in numerous products marketed as dietary supplements. As such, a USP monograph for GABA is under evaluation [13]. Manufacturers recommend GABA intake in the range of 1.5–3000 mg/day, although for the majority of products, recommended intake is 100 mg/daily. The NNHPD monograph for Cognitive Function Products recommends a daily intake of 50–3000 mg GABA that does not exceed 750 mg per single dose (NNHPD).

Glutamic acid or glutamate is synthesized from *α*-ketoglutaric acid by mitochondrial glutamate dehydrogenase [14]. Glutamate is also synthesized from glutamine by glutaminase in the central nervous system. It functions as a neurotransmitter and an intermediate in many fundamental biochemical reactions. In addition, Glu acts as a precursor for the inhibitory neurotransmitter GABA [14].

Glu and GABA have been found in various plant species. The richest sources of these amino acids are cereals (brown rice germ, brown rice sprouts, barley sprouts, bean sprouts, beans, corn, barley, and brown rice), vegetables (spinach, broccoli, onion, potatoes, sweet potatoes), fruits (squash, apples, blueberries) and some herbs (chamomile, salvia, lavender) [9,15,16]. Lactic acid bacteria also produce GABA during fermentation, thus enriching fermented food with this bioactive amino acid [6]. To evaluate the Glu and GABA content of foods, it is important to have a simple and reliable quantitative analytical method. Due to the lack of chromophore groups, it is difficult to detect these amino acids with UV-Vis and fluorescence detectors. To overcome this drawback, pre-column derivatization is required, and a variety of reagents have been used for the purpose [17]. The most commonly used derivatizing agents are *o*-phthalaldehyde, dansyl chloride, 2-hydroxynaphthaldehyde, 9-fluorenylmethyl chloroformate, etc. [18,19,20,21]. It should be noted that their usage is related to some disadvantages. For example, derivatives obtained by o-phthalaldehyde are unstable at room temperature, and derivatization with 2-hydroxynaphthaldehyde requires high temperature, whereas 9-fluorenylmethyl chloroformate causes interference due to its insolubility and hydrolysis products.

The aim of the current work was to validate an HPLC method for GABA and Glu determination and to apply it to the analysis of these amino acids in commonly consumed fruits, vegetables, cereals, pseudocereals, nuts, and medicinal plants. Due to the simple and fast sample preparation, stable products, and rapid derivatization, dansyl chloride was used as a derivatizing agent for Glu and GABA.

## 2. Results and Discussion

### 2.1. Method Validation

**Figure 1:**
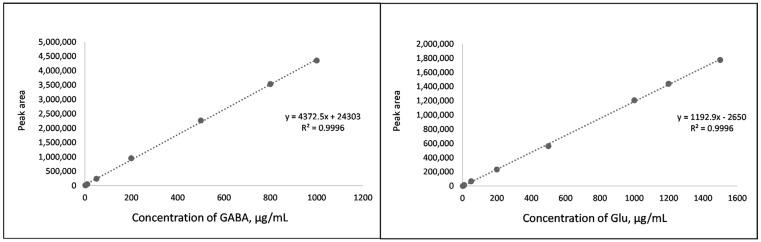
Standard curves for GABA and Glu.

Several analytical characteristics (linearity range, LOD, LOQ, accuracy, and precision) are required for the development and validation of any chromatographic method. In our study, the linearity was evaluated using calibration curves prepared with Glu and GABA standard solutions. Standard curves were constructed using the ratio between peak areas and concentrations of each analyte. Linearity was observed in the range from 2 μg/mL to 1500 μg/mL for Glu and from 2 μg/mL to 1000 μg/mL for GABA. Correlation coefficients for both standards were R^2^—0.9996, and standard curves are shown in Figure 1.

LOD and LOQ for GABA and Glu were calculated from the linearity data using a residual standard deviation of the regression lines and were found to be 0.60 μg/mL and 1.98 μg/mL, and 0.35 μg/mL and 1.15 μg/mL for Glu and GABA derivatives, respectively. Given the extraction conditions and plant material to extragent ratio of 1:15, LOD and LOQ were equivalent to 0.90 mg and 2.97 mg Glu, and 0.53 mg and 1.73 mg GABA in 100 g dried plant material. These low detected concentrations show that the method is sensitive.

To test the accuracy of the extraction method, a recovery experiment was performed with a derivatized extract from freeze-dried potato. For this purpose, Glu and GABA concentrations were analyzed and calculated in potato extract using the calibration curves depicted in Figure 1. After that, potato extract was spiked with 100% and 50% of its native Glu and GABA contents. As shown in Table 1 and Table 2, the percent recoveries of Glu and GABA were in the range of 97.8–103.2% and 102.6–104.9%, respectively, demonstrating an acceptable accuracy of the analytical method.

The reliability and reproducibility of the method were evaluated by determining the inter-day and intra-day accuracy and precision. The precision of the GABA and Glu extraction procedure was calculated as the %RSD, which was 2.73% and 0.55%, respectively, for ten replicate extractions (Table 3).

The results for inter-day and intraday precision and accuracy of the method are summarized in Table 4.

There was a very good separation between the two individual amino acids, as demonstrated in Supplementary Figure S1, showing an analysis chromatogram of potato extract. These results show that the method is selective and specific enough to separate and quantify Glu and GABA from complex mixtures like vegetable extracts.

### 2.2. Content of GABA and Glu in Plant Foods

**Figure 2:**
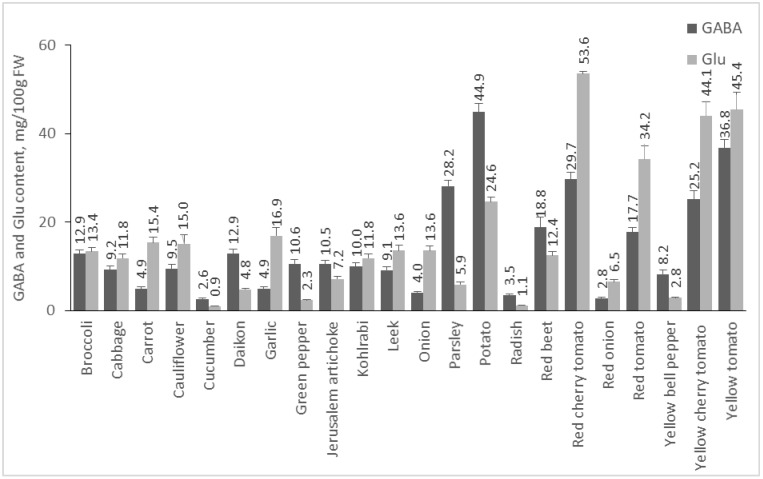
GABA and Glu content of vegetables. Results are presented as mean values ± standard deviation (SD).

**Figure 3:**
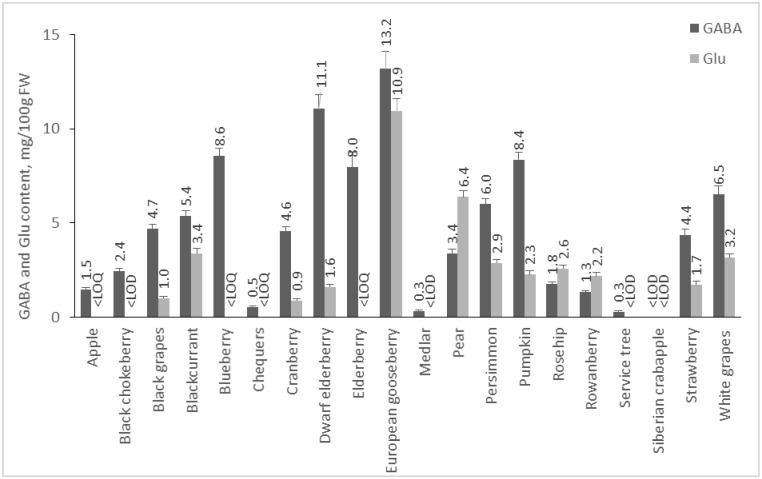
GABA and Glu content of fruits. Results are presented as mean values ± standard deviation (SD).

**Figure 4:**
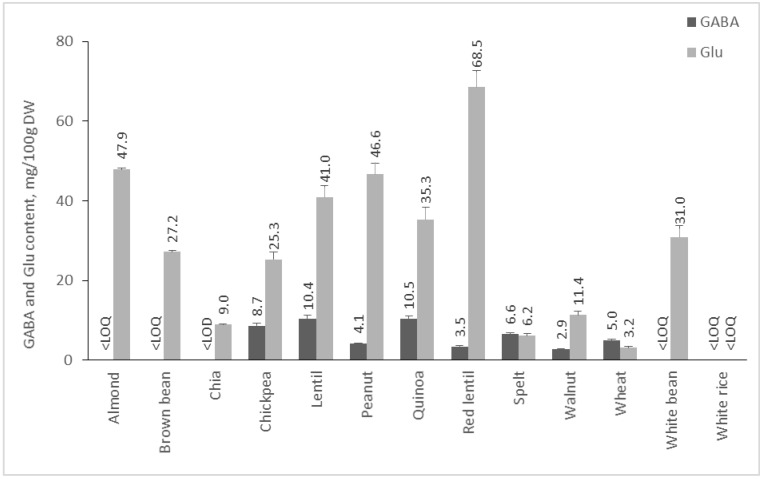
GABA and Glu content of cereals, pseudocereals, nuts, and legumes. Results are presented as mean values ± standard deviation (SD).

**Figure 5:**
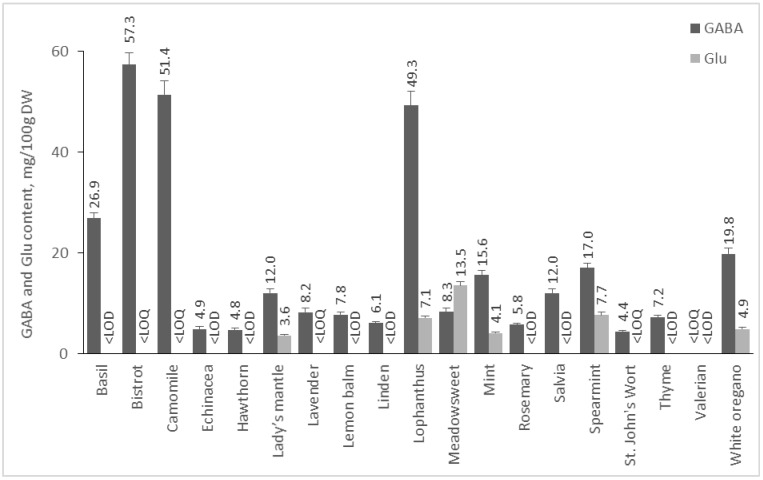
GABA and Glu content of medicinal plants. Results are presented as mean values ± standard deviation (SD).

The amount of Glu and GABA in plants depends on many factors, including species, environmental conditions, post-harvest treatments, etc. In addition, it is known that in response to stress factors, such as hypoxia, cold shock, and mechanical stimulation, the amount of GABA increases [22,23]. In our study, we analyzed 55 plant foods, including fruits, vegetables, cereals, pseudocereals, and legumes, and 19 medicinal plants for their GABA and Glu contents. Results are presented in Figure 2, Figure 3, Figure 4 and Figure 5.

#### 2.2.1. Content of GABA and Glu in Vegetables

From the investigated plant materials, the group of vegetables is the richest dietary source of GABA and Glu (Figure 2). Potato, the vegetable in which GABA was found for the first time [24], contained the highest quantity of GABA (44.86 mg/100 g FW), which is in agreement with previous studies (6–61 mg/100 g FW), [25]. Tomatoes are a rich source of these amino acids, as well. Some studies reported that the amount of GABA varies in the range of 8.8–189.7 mg/100 g FW [26]. Our results show that the quantity of GABA is 36.82 mg/100 g FW in yellow cherry tomato, 29.67 mg/100 g FW in red cherry tomato, 25.16 mg/100 g FW in yellow cherry tomato, and 17.73 mg/100 g FW in red tomato. The red cherry tomato is the vegetable with the highest concentration of Glu (53.58 mg/100 g FW), followed by yellow tomato and yellow cherry tomato. Parsley is also high in GABA with 28.18 mg/100 g FW and ranks 4th among the investigated vegetables. Data on the availability of GABA in parsley were not found in the literature. Beetroot is also a rich source of GABA with 18.84 mg/100 g FW. Other studies report GABA content in red beetroot 16 mg/100 g FW [27]. Park et al. presented results for the presence of GABA in different types of green cabbage, with the content varying in the range of 3.2–7.1 mg/100 g FW [28]. In our study, cabbage contained 9.18 mg/100 g FW GABA. In broccoli, GABA was 12.88 mg/100 g FW, and a previous publication reported a result of 2 mg/100 g FW [29]. Carrots are an example of a root vegetable that is low in GABA. Oh et al. reported 0.3 mg/100 g DW in carrot [9], whereas in our analysis, the amount of this amino acid was 4.88 mg/100 g FW. Radishes were reported to contain 28 mg/100 g DW [30], while the radishes in our study contained 3.47 mg/100 g FW. Onions and garlic are relatively poor in GABA (4.03 and 4.93 mg/100 g FW). Other authors reported GABA content in onions of 0.1 mg/100 g DW [9].

#### 2.2.2. Content of GABA and Glu in Fruits

Fruits are not as rich in GABA and Glu as the investigated vegetables, but the amount of GABA in them prevails in comparison to Glu (Figure 3). European gooseberry contains the highest amounts of GABA and Glu—13.18 and 10.95 mg/100 g FW. Dwarf elderberry, blueberry, and pumpkin are other fruits with high content of GABA—11.06, 8.56, and 8.37 mg/100 g FW, respectively. Zhang et al. [15] reported a similar result for blueberries—8.9 mg/100 g FW GABA. To our knowledge, there is no data in the available literature for GABA content of European gooseberry, black elderberry, pumpkin, rowanberry, and cranberry. In our study, the GABA content of white grapes (var. Tsarica) was 6.51 mg/100 g FW, and that of investigated black grapes was 4.7 mg/100 g FW, which is comparable to the published data for ‘Magnolia’ white grapes (7.4 mg/100 g FW) [31]. The content of this amino acid has been reported in different Merlot wine varieties, with the amount varying according to ripening (green: 8.0 mg/100 g, transient 7.0 mg/100 g, and purple 4.0 mg/100 g [32]. Deewatthanawong, Nock & Watkins investigated several strawberry cultivars for the GABA content, reporting values of 1.5 to 3.5 mg/100 g [33], which is comparable to our result—4.36 mg/100 g FW. Different cultivars of apples were also studied, and the amount of GABA varied depending on the variety. In “Honeycrisp,” GABA was 10.0 mg/100 g FW [34], and in “Jonagored,” 0.3 mg/100 g FW [33].

#### 2.2.3. Content of GABA and Glu in Cereals, Pseudocereals, Nuts, and Legumes

From the literature, it is known that legumes are rich in GABA. However, the present study observed that the quantity of Glu in the analyzed cereals, pseudocereals, nuts, and legumes is much higher than GABA content (Figure 4). From analyzed plant materials in this group, red lentils had the highest content of Glu (68.54 mg/100 g DW), whereas quinoa is the pseudocereal with the highest quantity of GABA—10.45 mg/100 g DW. Other studies have shown that the amount of GABA in quinoa varies from 10 to 70 mg/100 g DW, depending on the variety [35], whereas glutamic acid content of 48–200 mg/100 g DW in different types of lentils has been reported [36]. GABA content of lentils has been reported in the range from 4 to 40 mg/100 g DW [37,38], which complies with our result—10.37 mg/100 g DW. Although GABA has been reported in substantial amounts in beans [9], in our study, its content in the two analyzed varieties was below the LOQ of the validated HPLC method.

As already stated, glutamic acid content was significantly higher in comparison to GABA content, and even more in the majority of the samples in this group, GABA content was below the LOQ of the method. This is interesting because all samples in this group are seeds or nut kernels whose purpose is to germinate, creating a new plant. Given the versatile role of GABA in plants [39], seeds and nut kernels could be assumed as a Glu depot. Glu could be transformed into GABA after germination, which could elevate plant stress tolerance, improve photosynthesis, inhibit reactive oxygen species generation, activate antioxidant enzymes, and regulate stomatal opening in drought stress. It has been observed that GABA increases significantly after germination and soaking cereals like beans, rice, and wheat [40,41]. For example, an increase in the GABA content of chickpeas from 6.42 mg/100 g to 245.76 mg/100 g after germination was observed [42]. Therefore, it would be of interest to investigate the glutamate decarboxylase activity of seeds and nuts and the opportunity for the development of GABA-enriched nutraceuticals from germinated seeds of cereal, pseudocereal, and legume cultures.

#### 2.2.4. Content of GABA and Glu in Medicinal Plants

The available information on the presence of GABA in medicinal plants is scarce [16], and a current study reports the content of GABA in several herbs for the first time. On the contrary to seeds and nuts, the content of GABA in dried herbs prevailed significantly over that of Glu, and the latter was below the LOQ in the majority of the investigated herbs (Figure 5). Bistrot (*Polygonum bistorta* L.) roots were the richest source of GABA (57.3 mg/100 DW), and this is the first report for the GABA content of that herb. Chamomile flowers (51.4 mg/100 g DW), lophantus (49.3 mg), and basil (26.9 mg/100 g are rich sources of GABA, as well. It is interesting that anxiolytic effects have been reported for chamomile, basil, and white oregano, but this effect was attributed to flavonoids (apigenin) in the case of chamomile [43] or some phenolic constituents from the essential oil for the other herbs [44,45]. It is already known that natural products and, in particular, some phenolic compounds act as GABA receptor modulators [46]. In addition, it would be of particular interest to investigate whether GABA contributes to the observed effects. Interestingly, the content of GABA in these herbs is higher or comparable to several varieties of green, white, or black tea (*Camelia sinensis*), however without reaching that of the specially designed GABA tea, with a GABA content of a minimum of 150 mg/100 g DW [47].

## 3. Materials and Methods

### 3.1. Chemicals and Reagents

GABA, Glu, dansyl chloride, and sodium acetate were purchased from Sigma-Aldrich Chemical Co. Ltd. (St Louis, MO, USA). Methanol and acetone were obtained from Fisher Scientific UK Ltd. Ethanol and sodium hydrogencarbonate were delivered from a local distributor from Sofia, Bulgaria. Demineralized water was used for the experiments.

### 3.2. Plant Materials

Fresh fruits and vegetables, dried cereals, pseudocereals, nuts, and legumes (shown in Supplementary Table S1) were purchased from local markets in Plovdiv, Bulgaria. All fresh materials were frozen and freeze-dried in Alpha 1–4 LDplus laboratory freeze dryer (Martin Christ Gefriertrocknungsanlagen GmbH, Osterode am Harz, Germany) and powdered before extraction, derivatization, and HPLC analysis.

### 3.3. Preparation of Extracts

Plant materials were grounded to fine powder immediately prior to extraction and analysis. For the extraction, 1 g of powdered sample was mixed with 15 mL of 75% ethanol and extracted on a magnetic stirrer for 1 h at room temperature. The extracts were filtered, and supernatants were collected for derivatization and HPLC analysis.

### 3.4. Derivatization of GABA and Glu

Derivatization of GABA and Glu was based on the procedure described by Gong et al. [48] with some modifications. HPLC derivatizing reagent was prepared by mixing 5 mg dansyl chloride with 10 mL acetone. Standard solutions of GABA and Glu were prepared with ultra-pure water. For the derivatization, 100 μL of the sample (GABA and Glu standard solutions or extract) were mixed with 900 µL sodium hydrogencarbonate buffer (0.1 M, pH = 8.7) and 1000 µL dansyl chloride solution to a final volume of 2 mL. The solution was homogenized through vortexing and heated for 1 h at 55 °C. After that, the solution was cooled down to room temperature and filtered through a 0.45 µm PTFE syringe filter (Filtratech, Saran, France) before HPLC analysis.

### 3.5. HPLC Determination of GABA and Glu Derivatives

HPLC analyses were performed on the UHPLC system Nexera-i LC2040C Plus (Shimadzu Corporation, Kyoto, Japan) with a UV-VIS detector and a binary pump. The column was Poroshell 120 EC-C18 (3 mm × 100 mm, 2.7 μm), thermostated at 30 °C. The flow rate was 0.3 mL/min, and the injection volume was 5 μL. The detection of the derivatives was made at λ = 254 nm. The mobile phase consisted of A: methanol and B: 900 mL 0.05M sodium acetate: 100 mL methanol (pH = 8). The gradient condition started with 20% A, between 0 min and 10 min, linearly increased to 60% A and then to 100% A at 11 min. From 12 to 17 min, continued isocratic with 100% A, and after that, from 18 min to 25 min, A linearly decreased to 20%.

### 3.6. HPLC Validation

The analytical method was validated for linearity range, the limit of detection (LOD), the limit of quantitation (LOQ), accuracy, and precision according to ICH harmonized tripartite guidelines [49,50].

#### 3.6.1. Linearity

The linearity of the calibration curves was constructed by analysis of a series of triplicate injections of standard solutions at different concentrations. It is determined by the point of intersection of the calibration curve, slope, and regression coefficient (R^2^). Calibration curves for each standard compound were obtained by plotting the peak areas against the corresponding concentrations of Glu and GABA, and results were used to calculate linearity [49].

#### 3.6.2. Limit of Detection (LOD) and Limit of Quantification (LOQ)

A series of decreasing concentrations of standard solutions were analyzed to determine the LOD and LOQ. The LOD is the lowest concentration of the sample that can be detected under the method’s conditions, and the LOQ is the lowest concentration of a sample that could be quantified. These parameters were calculated from the signal-to-noise ratio using the following equations: [50]

$$LOD=\frac{3.3\times SD–intercepts\;of\;regression\;lines}{slope}$$

$$LOQ=\frac{10\times SD–intercepts\;of\;regression\;lines}{slope}$$

#### 3.6.3. Recovery

The recovery of the analytical method was determined by the standard addition method with a freeze-dried potato sample spiked with 100% and 50% of its Glu and GABA contents. Recovery of Glu and GABA was calculated by the following equation, and the acceptance criteria of the percent recovery were 90–107% [50].

(1) $$Recovery\;\left(\%\right)=100\;\times \;\frac{\left(amount\;of\;Glu/GABA\;in\;spiked\;sample-amount\;of\;Glu/GABA\;in\;unspiked\;sample\right)}{amount\;of\;Glu/GABA}$$

#### 3.6.4. Precision

The precision of the analytical method was determined by inter and intraday accuracy. The repeatability of the extraction process (analysis repeatability) was determined by tenfold extraction of Glu and GABA from 1g freeze-dried potato on the same day, under the stated experimental condition and analysis. The intermediate precision of the method was assessed by carrying out the analysis on different days. The standard deviation (SD) and relative standard deviation (RSD) were calculated for each day [50].

### 3.7. Statistical Analysis

All extractions were performed twice, and HPLC analysis of each extract was performed in duplicate. The results are expressed as mean values ± SD (*n* = 4). MS Office Excel 2016 was used for the statistical analysis.

## 4. Conclusions

In the current study, a quick and selective method for the concomitant determination of gamma-aminobutyric acid and glutamic acid in plant foods and medicinal plants was validated. The method was successfully used to determine GABA and GA contents in 55 plant foods, including fruits, vegetables, cereals, pseudocereals, nuts and legumes, and 19 medicinal plants. The obtained results enrich the available information on the content of gamma-aminobutyric and glutamic acids in plant foods and could be used for the development of GABA-enriched functional foods. Vegetables, particularly potatoes and different tomato varieties, are the most important dietary source of GABA. Fruits are relatively low in GABA; however, they could be used for the development of fermented nutraceuticals with elevated GABA content. GABA is either absent or very low in nuts and seeds of cereals, pseudocereals, and legumes. However, these foods are a rich source of Glu, which could be transformed into GABA after germination, opening the possibilities elaboration of GABA-enriched nutraceuticals from germinated seeds. Medicinal plants are yet underestimated and barely researched sources of GABA. Even though some of the investigated herbs contain relatively high amounts of GABA, their contribution to the daily GABA intake would be limited due to the low quantities consumed as herbal infusions or extracts. However, herbs could be used for the fortification of fermented foods/beverages with natural antioxidants and other compounds with health benefits [51].

## Figures and Tables

**Table 1:** Repeatability and accuracy for Glu in potato (*n* = 5).

Added Glu, (mg)	Recovered Glu, (mg)	Recovery, (%)	Mean, (%)	RSD, (%)
1.1	1.07	97.2	97.8	0.68
1.1	1.08	97.8		
1.1	1.08	98.0		
1.1	1.09	98.8		
1.1	1.07	97.1		
0.5	0.52	103.3	103.2	3.29
0.5	0.50	99.4		
0.5	0.54	108.6		
0.5	0.51	101.7		
0.5	0.51	102.7		

**Table 2:** Repeatability and accuracy for GABA in potato (*n* = 5).

Added GABA, (mg)	Recovered GABA, (mg)	Recovery, (%)	Mean, (%)	RSD, (%)
1.8	1.91	106.2	102.6	3.72
1.8	1.88	104.3		
1.8	1.73	96.4		
1.8	1.88	104.4		
1.8	1.83	101.8		
0.9	0.95	105.6	104.9	0.73
0.9	0.95	105.2		
0.9	0.95	105.3		
0.9	0.93	103.7		
0.9	0.94	104.8		

**Table 3:** Repeatability of GABA and Glu extraction (*n* = 10).

Sample №	Sample Mass, (g)	Extracted GABA, (mg/g)	Extracted Glu, (mg/g)
1	1.003	1.76	1.09
2	1.002	1.77	1.09
3	1.008	1.74	1.09
4	1.009	1.74	1.10
5	1.027	1.81	1.08
6	1.017	1.83	1.09
7	1.030	1.85	1.09
8	1.021	1.85	1.08
9	1.032	1.87	1.10
10	1.031	1.86	1.10
MEAN	-	1.81	1.09
SD	-	0.049	0.006
RSD	-	2.73	0.55

**Table 4:** Precision (intra-day and inter-day) of HPLC analysis of GABA and Glu (*n* = 5).

Day	Added GABA, (mg)	Recovered GABA, (mean ± SD), (mg)	Precision, (RSD)	Accuracy, (% recovery)
1	1.8	1.85 ± 0.07	3.72	102.6
	0.9	0.94 ± 0.01	0.73	104.9
2	1.8	1.83 ± 0.05	2.55	101.7
	0.9	0.91 ± 0.04	4.64	101.4
**Day**	**Added Glu, (mg)**	**Recovered Glu,** **(mean ± SD) (mg)**	**Precision,** **(RSD)**	**Accuracy,** **(% recovery)**
1	1.1	1.08 ± 0.01	0.68	97.8
	0.5	0.52 ± 0.02	3.29	103.2
2	1.1	1.05 ± 0.02	2.24	95.8
	0.5	0.51 ± 0.02	4.83	101.4

## Data Availability

Data is contained within the article and Supplementary Materials.

## Supplementary Materials

The following supporting information can be downloaded at: https://www.mdpi.com/article/10.3390/molecules28010084/s1, Table S1: Plant materials analyzed for gamma-aminobutyric acid and glutamic acids, Figure S1: Chromatogram with separation of gamma-aminobutyric acid and glutamic acids in an extract from freeze-dried potatoes: Rt (Glu)—5.831 min; Rt (GABA)—9.645 min.
