# Supplementary material for: Validation of HPLC Method for Analysis of Gamma-Aminobutyric and Glutamic Acids in Plant Foods and Medicinal Plants
Source: Molecules. 2022 Dec 22;28(1):84. doi: 10.3390/molecules28010084 (PMC9822420; doi:10.3390/molecules28010084)
Supplement: Supplementary file 1 [file molecules-28-00084-s001.zip › molecules-2100867-supplementary.pdf]

Supplementary Table S1. Plant materials analyzed for gamma-aminobutyric acid and glutamic acids

**Plant material**	**Latin name**	**Botanical family**
Fruits		
Apple	*Malus domestica*	Rosaceae
Black chokeberry	*Aronia melanocarpa*	Rosaceae
Blackcurrant	*Ribes nigrum*	Grossulariaceae
Black grapes	*Vitis vinifera*	Vitaceae
Blueberry	*Vaccinium uliginosum*	Ericaceae
Chequers	*Sorbus torminalis*	Rosaceae
Cranberry	*Vaccinium vitis-idaea*	Ericaceae
Dwarf elderberry	*Sambucus ebulus*	Adoxaceae
Elderberry	*Sambucus nigra*	Adoxaceae
European gooseberry	*Ribes uva-crispa*	Grossulariaceae
Medlar	*Mespilus germanica*	Rosaceae
Pear	*Pyrus communis*	Rosaceae
Persimmon	*Diospyros kaki*	Ebenaceae
Pumpkin	*Cucurbita pepo*	Cucurbitaceae
Rosehip	*Rosa canina*	Rosaceae
Rowanberry	*Sorbus aucuparia*	Rosaceae
Service tree	*Sorbus domestica*	Rosaceae
Siberian crabapple	*Malus baccata*	Rosaceae
Strawberry	*Fragaria ananassa*	Rosaceae
White grapes	*Vitis vinifera*	Vitaceae
Vegetables		
Broccoli	*Brassica oleracea var. italica*	Brassicaceae
Cabbage	*Brassica oleracea*	Brassicaceae
Carrot	*Daucus carota*	Apiaceae
Cauliflower	*Brassica oleracea var. botrytis*	Brassicaceae
Cucumber	*Cucumis sativus*	Cucurbitaceae
Daikon	*Raphanus raphanistrum subsp. sativus*	Brassicaceae
Garlic	*Allium sativum*	Amaryllidaceae
Green pepper	*Capsicum annuum 'Baron'*	Solanaceae
Jerusalem artichoke	*Helianthus tuberosus*	Asteraceae
Kohlrabi	*Brassica oleracea var. gongylodes*	Brassicaceae
Leek	*Allium porrum*	Alliaceae
Onion	*Allium cepa*	Amaryllidaceae
Parsley	*Petroselinum crispum*	Apiaceae
Potato	*Solanum tuberosum*	Solanaceae
Radish	*Raphanus sativus*	Brassicaceae
Red beet	*Beta vulgaris*	Amaranthaceae
Red cherry tomato	*Solanum lycopersicum var. cerasiforme*	Solanaceae
Red onion	*Allium cepa*	Amaryllidaceae
Red tomato	*Solanum lycopersicum*	Solanaceae
Yellow bell-pepper	*Capsicum annuum 'Baron'*	Solanaceae
Yellow cherry tomato	*Solanum lycopersicum*	Solanaceae
Yellow tomato	*Solanum lycopersicum*	Solanaceae
Cereals, pseudocereals, legumes and nuts		
Quinoa	*Chenopodium quinoa*	Amaranthaceae

White rice	*Oryza sativa*	Poaceae
Chia	*Salvia hispanica*	Lamiaceae
Wheat	*Triticum aestivum*	Poaceae
Spelt	*Triticum monococcum*	Poaceae
Chickpea	*Cicer arietinum*	Fabaceae
Lentil	*Lens culinaris*	Fabaceae
Red lentil	*Lens culinaris*	Fabaceae
Brown bean	*Phaseolus vulgaris*	Fabaceae
Colorful bean	*Phaseolus vulgaris*	Fabaceae
Peanut	*Arachis hypogaea*	Fabaceae
Walnut	*Juglans regia*	Juglandaceae
Almond	*Prunus dulcis*	Rosaceae
Medicinal plants		
Basil, leaf	*Ocimum basilicum*	Lamiaceae
Bistort, root	*Bistorta officinalis*	Polygonaceae
Chamomile, flower	*Matricaria chamomilla*	Asteraceae
Echinacea, root	*Echinacea purpurea*	Asteraceae
Hawthorn, leaf and flower	*Crataegus Monogyna*	Rosaceae
Lady's mantle, aerial parts	*Alchemilla vulgaris*	Rosaceae
Lavender, flower	*Lavandula angustifolia*	Lamiaceae
Lemon balm, leaf	*Melissa officinalis*	Lamiaceae
Linden, flower	*Tilia cordata*	Malvaceae
Lophanthus, aerial parts	*Lophanthus chinensis*	Lamiaceae
Meadowsweet, aerial parts	*Filipendula ulmaria*	Rosaceae
Mint, leaf	*Mentha piperita*	Lamiaceae
Rosemary, aerial parts	*Rosmarinus officinalis*	Lamiaceae
Salvia, leaf	*Salvia officinalis*	Lamiaceae
Spearmint, leaf	*Mentha spicata*	Lamiaceae
St. John's Wort, aerial parts	*Hypericum perforatum*	Hypericaceae
Thyme, aerial parts	*Thymus vulgaris*	Lamiaceae
Valerian, roots	*Valeriana officinalis*	Valerianaceae
White oregano, leaf	*Origanum heracleoticum*	Lamiaceae

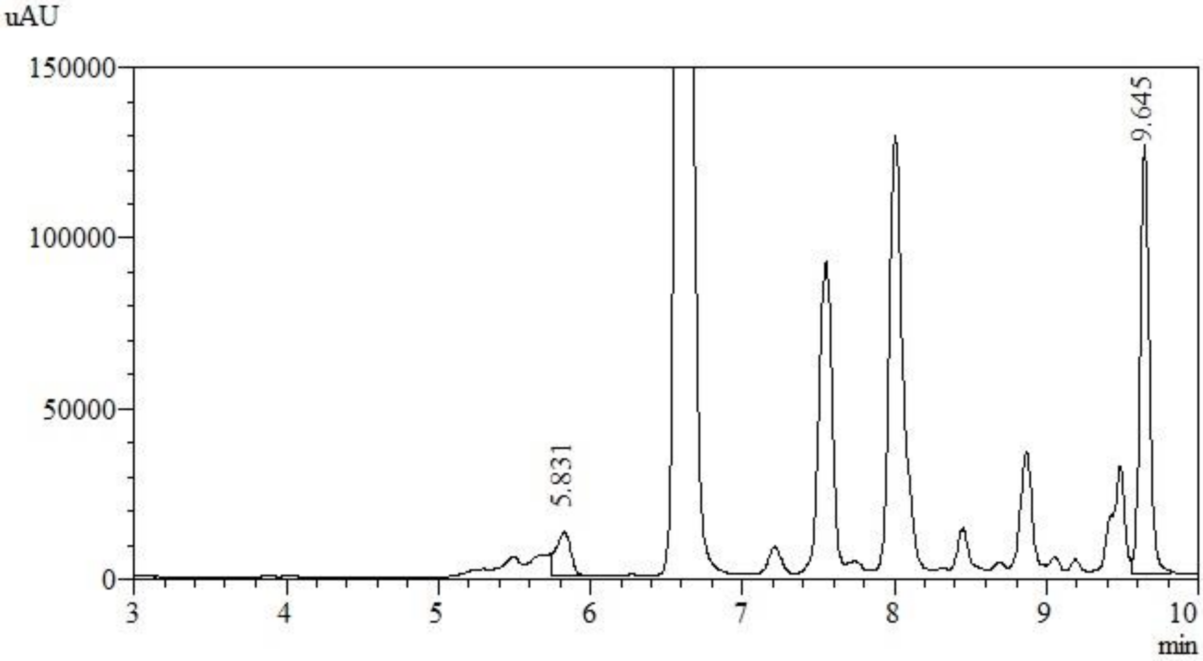

Supplementary Figure S1. Chromatogram with separation of gamma-aminobutyric acid and glutamic acids in extract from freeze-dried potatoes: Rt (Glu) - 5.831 min; Rt (GABA) – 9.645 min.
